# Immunomodulation on tumor immune microenvironment in acquired targeted therapy resistance and implication for immunotherapy resistance

**DOI:** 10.1016/j.tranon.2025.102353

**Published:** 2025-03-08

**Authors:** Ming-Yu Chou, Muh-Hwa Yang

**Affiliations:** a Department of Medical Education, Taipei Veterans General Hospital, Taipei 112201, Taiwan; b Institute of Clinical Medicine, National Yang Ming Chiao Tung University, No. 155, Sec. 2, Li-Nong Street, Taipei 112304, Taiwan; c Cancer and Immunology Research Center, National Yang Ming Chiao Tung University, Taipei 112304, Taiwan; d Department of Oncology, Taipei Veterans General Hospital, Taipei 112201, Taiwan

**Keywords:** Targeted therapy resistance, Immunotherapy resistance, Tumor-immune microenvironment

## Abstract

The emergence of molecularly targeted therapies and immunotherapies has revolutionized cancer treatment, yet the optimal sequencing of these modalities remains debated. While targeted therapies often induce initial immunostimulatory effects, the development of resistance is accompanied by dynamic alterations in the tumor-immune microenvironment. These changes can promote tumor growth, hinder immune surveillance, and contribute to subsequent immunotherapy resistance. This review focuses on solid tumors and summarizes the immunomodulatory effects arising in the context of targeted therapy resistance, highlighting the challenges they pose for the subsequent immunotherapy efficacy.

## Highlights

• Acquired resistance to targeted therapy promotes immunosuppressive TIME remodeling.
• Clinical studies reveal optimal sequencing of targeted and immune therapies varies by cancer type.
• Cancer-immunity cycle guides development of resistance-overcoming strategies.

## Graphical abstract

**Figure:**
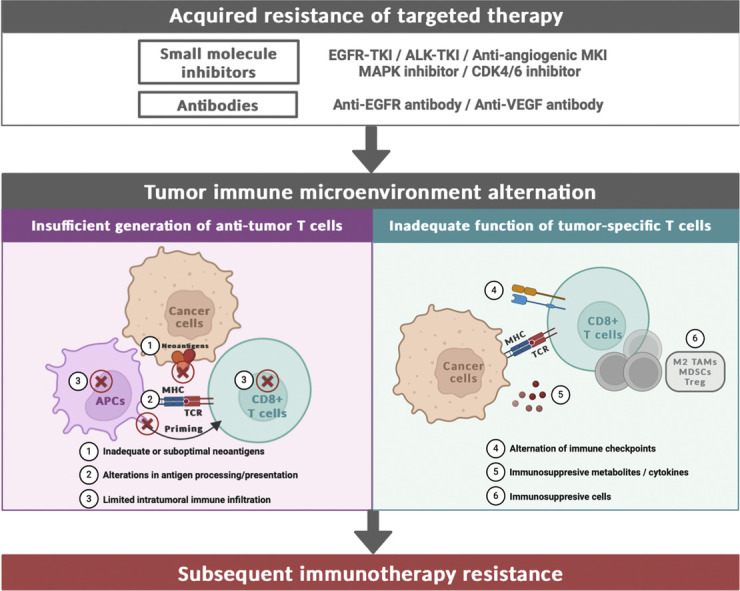

## Introduction

The advent of new-generation molecular targeted therapies and immunotherapeutic agents has revolutionized the landscape of cancer treatment. Molecular targeted agents are the cornerstone for precision medicine and offer spectacular tumor responses in oncogene-addicted cancers. Immunotherapies leverage immune system to eliminate cancer and can result in unprecedented long-term remissions. Both targeted therapies and immunotherapies are recommended as first-line treatments for various metastatic cancers such as melanoma and non-small cell lung cancer (NSCLC). However, due to increased toxicity when combined, these therapies are often administered sequentially to maximize patient benefit, leading to considerable debate over the preferred sequence of administration [1,2]. Despite recommendations in certain scenarios to begin with targeted therapy until resistance develops, followed by a switch to immunotherapy, clinical evidence indicates that patients who relapse after targeted therapy exhibit a lower overall response rate to immunotherapy compared to those who have not previously received targeted therapy [[3], [4], [5], [6]]. Nonetheless, how targeted therapy alters the tumor and its microenvironment in the context of resistance, and how these changes affect subsequent therapeutic responses, remain poorly understood.

A substantial body of preclinical and clinical data indicates that nearly all targeted agents exert immunomodulatory effects, both through direct interaction with cancer cells and by altering the function of immune cells [7]. Although the precise mechanisms by which immunomodulation arises from the inhibition of the intended molecular target in malignant cells remain to be elucidated, it is believed that targeted therapies generally mediate net immunostimulatory effects, at least during the initial phase of drug administration [8]. However, most tumors inevitably acquire resistance to targeted therapies, and during this progression, the dynamic alterations in both cancer cells and immune cells become pivotal questions to explore in depth. These changes may alter the composition of the tumor-immune microenvironment (TIME), and most importantly, which ensures the expansion of tumor, hampers the immune surveillance and further results in resistance to subsequent immune therapy.

In this review, we summarize the immunomodulatory effects on the TIME that occur in the context of resistance to targeted therapy and discuss how these changes may pose challenges for the efficacy of subsequent immunotherapies. Focusing primarily on solid tumors, where the TIME plays a more prominent role compared to hematopoietic malignancies [8,9], this review emphasizes melanoma, non-small cell lung cancer (NSCLC), and head and neck squamous cell carcinoma (HNSCC)—the cancer types most frequently studied in the context of sequencing with immune checkpoint inhibitors (ICIs). Other solid tumor types are mentioned briefly. Only targeted therapies relevant to ICI sequencing are covered in this article.

## Immunomodulatory effects in the context of acquired targeted therapy resistance

Targeted therapies primarily encompass small-molecule kinase inhibitors (SMKIs) and monoclonal antibodies (mAbs) [10]. SMKIs, by virtue of their small size, can target a wider range of intracellular and extracellular proteins, primarily by binding to the ATP-binding pocket of their target kinases [11]. In contrast, mAbs specifically target extracellular ligands (e.g., Bevacizumab against vascular endothelial growth factor [VEGF]), membrane receptors (e.g., Cetuximab against epidermal growth factor receptor [EGFR]), and membrane-bound proteins (e.g., Rituximab against CD20), exerting their effects through ligand blockade, receptor neutralization, or target internalization/degradation [11].

Despite the transformative impact of targeted therapies, tumors inevitably develop resistance. Mechanisms of resistance for both SMKIs and mAbs include alterations in target protein function (such as mutations, amplification, or loss of expression), activation of alternative signaling pathways, histological transformations (such as epithelial-to-mesenchymal transition or the transition from NSCLC to SCLC), and immune evasion as extensively reviewed [10,12]. Importantly, immune evasion not only contributes to the development of resistance to targeted therapies but may also arise as a consequence of therapeutic intervention, ultimately reducing the effectiveness of subsequent immunotherapies. The following sections will explore the complex immunomodulatory effects associated with acquired resistance to targeted therapies. The immunomodulatory effects of SMKIs and mAbs are summarized in Table 1.

**Table 1:** Summary of Immunomodulation of Small-Molecule Kinase Inhibitors and Antibodies.

**Blockage**	**Agents**		**Mechanism of action**	**Effects**	**Immunomodulation**					
					**Cancer cell - dependent**	**Immune cell - dependent**				
						**T cell**	**Macrophage**	**NK cell**	**DC**	**MDSCs**
**EGFR pathway**	TKI	Gefitinib	EGFR^WT^ATP-binding site blocker	Blocks downstream PI3K/AKT, RAS/MAPK, PLC-PKC and STAT pathways and inhibits survival, migration, cell cycle progression and angiogenesis	MHC class I & II ↑ PD-L1, CD47 ↓ NKAL ↑↓	CD8+ T recognition, accumulation and cytotoxicity ↑ Treg ↓	M2 TAM ↓	ADCC ↑↓	Phagocytosis ↑ Accumulation ↑	MDSC ↑
		Erlotinib	EGFR^WT^ ATP-binding site blocker		MHC class I & II ↑ NKAL ↑↓		MHC type I & II ↑	ADCC ↑↓	MHC type I & II ↑	
		Afatinib	ErbB (EGFR, HER2, HER4) family ATP-binding site blocker		MHC class I & II ↑					
		Osimertinib	EGFR T790M, L858R, and exon 19 deletion ATP-binding site blocker			CD8+ T recognition, accumulation ↑ Treg ↓	TAM ↓			MDSC ↑
	Antibody	Cetuximab	EGFR domain III monoclonal antibody		DAMP emission ↑		M2 to M1polarization ↑	ADCC ↑	Phagocytosis ↑	
**ALK pathway**	TKI	Crizotinib	ALK, HGFR, ROS1 ATP-binding site blocker	Blocks downstream PI3K/AKT, RAS/MAPK, PLC-PKC and STAT pathways and inhibits survival, proliferation, migration, and angiogenesis	DAMP emission ↑ HLA-I ↑			Infiltration ↑		
**Anti-angiogenic pathway**	TKI	Sunitinib	PDGFR a/b, VEGFR1/2/3,KIT, FTL3, CSF-1R and RET ATP binding site blocker	Blocks downstream PI3K/AKT, MAPK/ERK, Src Family Kinases, FAK pathways and inhibits survival, proliferation, migration, and angiogenesis	PD-L1 ↓	Treg activity ↓	TAM ↓			MDSC activity ↓
	Antibody	Bevacizumab	Serum VEGF-A monoclonal antibody		PD-L1 ↓	T_EFF_ activities↑ Treg ↓	TAM ↓		Maturation ↑	
**MAPK pathway**	BRAF TKI	Dabrafenib	BRAF ^WT, V600E, V600K, and V600D^ ATP-binding site blocker	Blocks downstream MEK/ERK activation and inhibits cell proliferation and survival	MHC class I, TAA ↑ PD-L1, IL8, VEGFA ↓					
		Vemurafenib	BRAF ^WT, V600E^ ATP-binding site blocker		TAA ↑ NKAL ↓					
	MEK TKI	Trametinib	MEK1 and MEK2 allosteric binding site blocker	Blocks downstream ERK activation and inhibits cell proliferation and survival	MHC class I, TAA, CXCL9 /CXCL10 ↑ PD-L1, IL8, VEGFA ↓	T_EFF_ function ↓				MDSC ↓
		Selumetinib	MEK1 and MEK2 allosteric binding site blocker			T_EFF_ function ↓				
**CDK4/6 pathway**	CDKI	Palbociclib	CDK ATP-binding site blocker	Blocks Rb/E2F pathway that drives G1 to S phase in the cell cycle and inhibit proliferation	MHC class I, TAA, CCL5, PD-L1 ↑	T_EFF_ activities↑ Treg ↓				
		Abemaciclib	CDK ATP-binding site blocker		MHC class I, B2M, type III IFN, SASP signatures, PD-L1↑	Treg ↓				

(ADCC: Antibody-Dependent Cell-Mediated Cytotoxicity; AKT: Protein Kinase B; ALK: Anaplastic Lymphoma Kinase; BRAF: B-Raf Proto-Oncogene Serine/Threonine Kinase; CCL5: C-C Motif Chemokine Ligand 5; CDKI: Cyclin-Dependent Kinase Inhibitor; CSF-1R: Colony Stimulating Factor 1 Receptor; CXCL10: Chemokine (C-X-C Motif) Ligand 10; CXCL9: Chemokine (C-X-C Motif) Ligand 9; DAMP: Damage-Associated Molecular Pattern; DC: Dendritic Cell; EGFR: Epidermal Growth Factor Receptor; ERK: Extracellular Signal-Regulated Kinase; FTL3: Fms-Like Tyrosine Kinase 3; HGFR: Hepatocyte Growth Factor Receptor; HLA: Human Leukocyte Antigen; ICD: Immunogenic Cell Death; IL8: Interleukin 8; KIT: KIT Proto-Oncogene Receptor Tyrosine Kinase; MAPK: Mitogen-Activated Protein Kinase; MDSC: Myeloid-Derived Suppressor Cell; MEK: Mitogen-Activated Protein Kinase Kinase; MHC: Major Histocompatibility Complex; NK cell: Natural Killer Cell; NKAL: Natural Killer Activation Ligand; PD-L1: Programmed Death-Ligand 1; PDGFR: Platelet-Derived Growth Factor Receptor; PI3K: Phosphoinositide 3-kinase; PKC: Protein Kinase C; PLC: Phospholipase C;; RET: Rearranged During Transfection; ROS1: ROS Proto-Oncogene 1; Rb: Retinoblastoma Protein; SASP: Senescence-Associated Secretory Phenotype; STAT: Signal Transducer and Activator of Transcription; Src: (Refers to a family of kinases); TAA: Tumor-Associated Antigen; TAM: Tumor-Associated Macrophage; TEFF: T effector; TKI: Tyrosine Kinase Inhibitor; Treg: Regulatory T cell; VEGFA: Vascular Endothelial Growth Factor A; VEGFR: Vascular Endothelial Growth Factor Receptor; WT: Wild Type)

### Small-molecule kinase inhibitors

#### Receptor tyrosine kinase inhibitors

##### EGFR-TKI

EGFR-TKIs have significantly improved outcomes for patients with NSCLC with EGFR-activating mutations. During treatment response, various EGFR-TKIs, including afatinib, erlotinib, and gefitinib, have been shown to enhance MHC class I and II expression on tumor cells, facilitating CD8+ T cell recognition and cytotoxicity [13]. Erlotinib also upregulates MHC II on macrophages and dendritic cells (DCs) [14], while gefitinib enhances DC phagocytosis of tumor cells by the decrement of the "don't eat me" signal CD47 on malignant cells [15]. Interestingly, while erlotinib and gefitinib have been shown to induce NK-cell-mediated antibody-dependent cellular cytotoxicity (ADCC) of NSCLC cells through upregulation of NK-cell activating receptors [[16], [17], [18]], contradictory findings have also been reported [19]. Despite conflicting reports on NK cell activation, multiple EGFR-targeting TKIs consistently downregulate PD-L1 expression in NSCLC cells by inhibiting NF-κB and IL6 signaling [20,21]. Although evidence indicate that EGFR-TKIs exert immunostimulatory effects, and this is further supported by a study showing increased abundance of DCs and CD8+ effector T cells in the early stages of gefitinib or Osimertinib treatment in vivo*,* such effects appear to be temporary and are rapidly followed by myeloid-derive suppressor cell (MDSC)-driven immunosuppression [22].

**Fig. 1:**
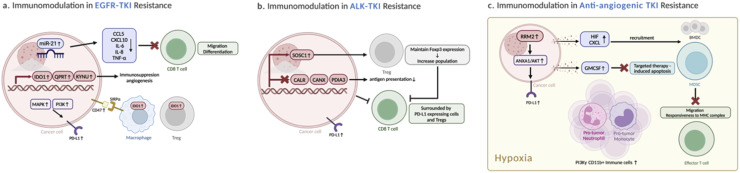
**Immunomodulation in Targeted Therapy Resistance – Tyrosine Kinase Inhibitors** (a) EGFR-TKI resistance fosters immunosuppression via upregulation of IDO and other kynurenine pathway genes, impacting T cells, myeloid cells, and angiogenesis. Increased IDO1-expressing macrophages and Tregs further contribute to immunosuppression. Additionally, miR-21 upregulation decreases pro-inflammatory cytokines, hindering CD8+ T cell function. Elevated CD47 expression impairs innate immunity. PD-L1 upregulation, mediated by MAPK, PI3K, and NF-kB, is also commonly observed. (b) ALK-TKI-resistant tumors demonstrate increased PD-L1 expression without a corresponding rise in CD8+ T cell cytotoxicity. Additionally, there's an upregulation of genes associated with Treg cell differentiation, and a downregulation of genes related to antigen presentation. (c) Chronic exposure to anti-angiogenic TKI creates a hypoxic environment, promoting the recruitment of BMDCs and MDSCs. In this setting, MDSCs evade apoptosis through tumor-released GM-CSF. Additionally, pro-tumor Gr1+ monocytes and neutrophils, along with PI3K-enriched CD11b+ immune cells, are also increased. Furthermore, RRM2 upregulation drives PD-L1 expression through the ANXA1/AKT signaling pathway. (AKT: Protein Kinase B; ALK-TKI: Anaplastic Lymphoma Kinase Tyrosine Kinase Inhibitor; ANXA1: Annexin A1; BMDCs: Bone Marrow-Derived Dendritic Cells; CD47: Cluster of Differentiation 47; CD8+ T cells: Cluster of Differentiation 8 positive T cells; EGFR-TKI: Epidermal Growth Factor Receptor Tyrosine Kinase Inhibitor; GM-CSF: Granulocyte-Macrophage Colony-Stimulating Factor; IDO: Indoleamine 2,3-dioxygenase; MAPK: Mitogen-Activated Protein Kinase; MDSCs: Myeloid-Derived Suppressor Cells; miR-21: microRNA-21; NF-κB: Nuclear Factor kappa-light-chain-enhancer of activated B cells; PI3K: Phosphoinositide 3-Kinase; PD-L1: Programmed Death-Ligand 1; RRM2: Ribonucleotide Reductase Subunit M2; Tregs: Regulatory T cells; TKI: Tyrosine Kinase Inhibitor; VEGF: Vascular Endothelial Growth Factor).

Several other significant alterations within the TIME have been reported in the context of EGFR-TKI resistance (Fig. 1). To investigate therapy-induced evolution in NSCLC, Maynard et al. employed single-cell RNA sequencing, uncovering significant upregulation of genes involved in the kynurenine metabolism pathway (IDO1, KYNU, QPRT) in EGFR-TKI-resistant tumors [23]. IDO1, as a rate-limiting enzyme in the kynurenine pathway, can influence diverse components of the TIME including T cell and myeloid cell populations as well as angiogenesis in favor of immunosuppression Interestingly, the study identified increased IDO1-expressing macrophages and T regulatory cells in resistant samples as well [24]. Additionally, in EGFR-TKI resistant cancer cells, upregulation of miR-21 reduces expression of CCL5, CXCL10, IL-6, IL-8, and TNF-α. This reduction leads to decreased migration of CD8+ T cells to tumor and impacts T cell differentiation [25]. Moreover, CD47 expression became up-regulated following in vitro drug resistance development and negatively affected innate immune surveillance [15].

PD-L1 overexpression is frequently observed in EGFR-TKI-resistant NSCLC cells [26,27]. This upregulation is mediated by the MAPK and PI3K pathways in several resistance mechanisms (c-MET amplification, HGF, and EGFR-T790M), with NF-κB specifically contributing to T790M-driven PD-L1 expression [26]. Another study revealed that the ERK1/2 pathway, rather than STAT3, has been associated with PD-L1 induction after resistant to gefitinib in cell line H1975 (EGFR L858R/T790M) and HCC827 (EGFR Del 19) in vivo*,* and significantly inhibits T-cell proliferation while slightly promoting apoptosis [27]. The above evidence suggests that EGFR-TKI therapy may confer a favorable TIME for subsequent ICI treatment. However, PD-1 inhibitors often yield unsatisfactory results in patients with high PD-L1 expression, likely due to immunosuppressive factors like increased regulatory T (Treg) cells and CD73 expression [28]. These findings underscore the complex immunomodulatory changes accompanying EGFR-TKI resistance and highlight the need for strategies to counteract immune evasion in resistant NSCLC.

In summary, EGFR-TKI resistance induces a complex immunosuppressive remodeling of the TIME, encompassing altered kynurenine metabolism, microRNA dysregulation, inhibition of phagocytosis, and PD-L1 upregulation via MAPK, PI3K, and NF-κB pathways. These changes compromise anti-tumor immunity and likely hinder subsequent ICI therapy, highlighting the need for strategies to overcome resistance-associated immune evasion.

##### ALK-TKI

In addition to EGFR mutations, anaplastic lymphoma kinase (ALK) gene rearrangements represent another key driver of NSCLC [29,30] . Approved ALK TKIs span three generations, with each demonstrating improved clinical efficacy, notably enhanced blood-brain barrier penetration [31]. The incidence of NSCLC amenable to ALK TKI therapy is low, given that only 4–5% of cases harbor ALK genetic rearrangements [29,30]. Consequently, discussions regarding the immunomodulatory effects of ALK TKIs remain scarce. Current evidence suggests that ALK-TKIs such as Crizotinib upregulate HLA-I expression through ALK-MAPK signaling in vitro [32] and enhance infiltration of cytotoxic NK CD56^dim^ cells (characterized by their nonspecific-antigen-dependent cell-killing ability) [33]. Furthermore, ALK-TKIs combined with chemotherapeutics induce damage-associated molecular pattern (DAMP) release, promoting immunogenic cell death [34].

From initial responsiveness to acquired resistance, dynamic shifts in immune cells and cytokines occur within the TIME (Fig. 1). Specifically, from baseline to the time of ceritinib-resistant, PD-L1 expression increased in both tumor and stromal compartments, while granzyme B production did not alter, implying lack of cytolytic function by CD8+ T cells [35]. These CD8+ T cells were often surrounded by PD-L1 expressing cells and FoxP3+ Treg cells, further supporting the notion that resistance may promote non-cytotoxic T cell accumulation [36]. Notably, the whole exome sequencing (WES) revealed no significant difference in tumor mutation burden between treatment-naive and resistant tumors. Additionally, T cell receptor sequencing showed no substantial changes in CD8+ T cell clonality or antigen specificity in resistant tumors. Gene set enrichment analysis (GSEA) revealed a marked upregulation of genes associated with Treg cell differentiation and immune suppression in resistant tumors. Notably, key genes involved in Treg function, such as SOCS1, were enriched, aligning with the observed increase in Treg cells in ceritinib-resistant tumors [36]. Similarly, single-sample GSEA (ssGSEA) from another study indicated a downregulation of antigen presentation genes, including CALR, CANX, and PDIA3, following chronic exposure to ALK inhibitors. Additionally, a reduced presence of neoantigens and a lower mutational load were noted as well [33] .

In conclusion, the development of ALK-TKI resistance is associated with increased PD-L1 expression in both tumor and stromal compartments, a functional deficit in CD8+ T cell cytolytic activity despite their presence, and a significant increase in Treg cells. These changes are accompanied by downregulation of antigen presentation genes and a reduction in neoantigen presence. Collectively, these resistance-associated alterations within the TIME likely contribute to immune evasion and may limit the effectiveness of subsequent immunotherapies.

##### Anti-angiogenic multi-kinase inhibitors

Most TKIs targeting angiogenesis are multi-kinase inhibitors (MKIs) that typically inhibit vascular endothelial growth factor receptor (VEGFR), platelet-derived growth factor receptor (PDGFR), fibroblast growth factor receptor (FGFR), c-kit, and rearranged during transfection [37]. Examples includes sunitinib, sorafenib, pazopanib, axitinib, cabozantinib, and lenvatinib, commonly used in metastatic renal cell carcinoma (mRCC), metastatic hepatocellular carcinoma (mHCC), and metastatic thyroid cancer [38].

Anti-angiogenic therapy effectively inhibits tumor growth by reducing vessel density yet could also create a corresponding hypoxic environment. Hypoxia could persist throughout the entire adaptation to anti-angiogenic therapy, leading to the expression of hypoxia-inducible factors (HIFs) and CXCL chemokines, which enhance the recruitment of MDSCs and other bone marrow-derive cells (BMDCs) to the tumor microenvironment and are associated with therapeutic resistance [39,40] (Fig. 1). Resistance is more likely a result of MKI-induced hypoxia, an inevitable consequence of chronic exposure to anti-angiogenic agents, rather than being driven by mechanisms regulated by immunosuppressive cells. In sunitinib-resistant RCC, persistently high levels of MDSCs were found due to escape from sunitinib-induced apoptosis, attributed to increased production of granulocyte-macrophage colony-stimulating factor (GM-CSF) [41]. These MDSCs produce nitric oxide, which reacts with superoxide, generating peroxynitrite (PNT). PNT nitrates T-cell receptors, reducing their responsiveness to antigen MHC complexes, and T-cell specific chemokines, blocking T-cell migration [42]. In response to the hypoxic environment and to reinitiate angiogenesis, sorafenib-resistant pancreatic neuroendocrine tumors in the Rip1Tag2 model exhibit increased infiltration of pro-tumor Gr1+ monocytes and neutrophils, along with PI3Kγ-enriched CD11b+ immune cells. Targeting Gr1+ cells was insufficient to sensitize angiogenic blockade because TAMs would compensate for the lack of such cells, leading to an oscillating pattern of distinct immune-cell populations [43]. Similarly, in another Rip1Tag2 model of pancreatic neuroendocrine tumors, VEGFR2 blockade by anti-VEGFR2 monoclonal antibody DC101 upregulated both ANG2 and TIE2 expression and enhanced the infiltration of TIE2-expressing macrophages [44]. TEMs, a subgroup of TAMs, suppress T cell proliferation, increase the ratio of CD4+ T cells to CD8+ T cells, and promote the expansion of FoxP3+ Treg cells [45]. Lymphangiogenesis often occurs in the late stage of resistance to anti-angiogenic therapies [46]. A study revealed that tumor-associated lymphatic vessels could upregulate PD-L1, reducing the stimulation of CD8+ T-cells by antigen-presenting lymphatic endothelial cells [47]. Apart from lymphangiogenesis-mediated PD-L1 upregulation, in sunitinib-resistant RCC, ribonucleotide reductase subunit M2 (RRM2) was upregulated and could also upregulate PD-L1 through the ANXA1/AKT signaling axis [48].

In summary, MKI-induced hypoxia inevitably drives MDSC and BMDC recruitment, MDSC survival via GM-CSF, and PNT-mediated T-cell inhibition. This resistance is further associated with increased pro-tumor monocytes, neutrophils, PI3Kγ-enriched immune cells, and TIE2-expressing macrophage infiltration, culminating in a complex immunosuppressive microenvironment.

#### Inhibitors targeting downstream signaling pathways

##### MAPK inhibitor

Given the frequency of aberrant MAPK/ERK activity observed in melanoma, several inhibitors have been developed to target components of this pathway, including inhibitors of BRAF, MEK and more recently RAS and ERK [49]. Interestingly, these agents not only harbor potent tumor-intrinsic effects, but also mediate a panel of therapeutically relevant immune-potentiating activity. Both BRAF and MEK inhibitors mediate various cancer-cell-dependent immunostimulatory effects, including upregulation of tumor associated antigens and improved antigen presentation [[50], [51], [52], [53], [54]], induction of immunogenic cell death [55], secretion of TH1 cytokines such as CXCL9 and CXCL10 [53] and downregulation of immunosuppressive factors, including IL8, VEGFA, and the MDSC chemoattractant SPP1 [54,56,57].

**Fig. 2:**
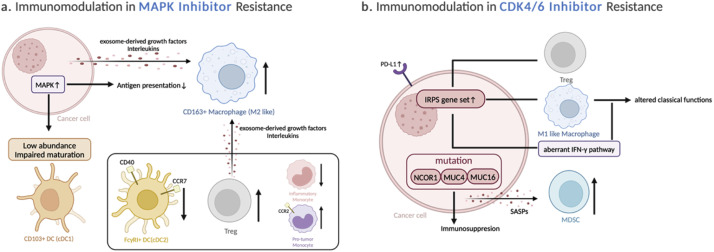
**Immunomodulation in Targeted Therapy Resistance – Inhibitors Targeting Downstream Signaling Pathways** (a) MAPKi resistance coincides with a decline in activated DCs, including cDC1 and cDC2 subsets, and a concomitant increase in Tregs and Ccr2+ monocytes. This impaired DC maturation is driven by reactivated MAPK signaling within tumor cells, rather than selective pressure from the immune response. Additionally, factors released by resistant cells promote the accumulation of immunosuppressive CD163+ M2 macrophages. (b) Resistance to CDK4/6 inhibitors is associated with IRPS gene set, relating to aberrant IFN signaling and Treg presence. Mutations in genes involved in immunosuppression were observed. Other immunomodulatory effects include PD-L1 elevation and SASP-induced MDSC recruitment. (CDK4/6: Cyclin-Dependent Kinase 4/6; DCs: Dendritic Cells; IFN: Interferon; IRPS: IFN-related Palbociclib-Resistance Signature; M2 Macrophage: Alternatively activated macrophages; MDSC: Myeloid-Derived Suppressor Cell; MAPKi: Mitogen-Activated Protein Kinase Inhibitor; PD-L1: Programmed Death-Ligand 1; SASP: Senescence-Associated Secretory Phenotype; Tregs: Regulatory T cells).

However, the immunosuppressive effects that emerge with resistance have been developed. BRAF inhibitors downregulate the expression of various NK-cell-activating ligands (NKALs) on the surface of melanoma cells, dampening NK cell attack [58]. Additionally, these inhibitors stimulate TAMs. For example, human melanoma cells that develop resistance to vemurafenib secrete higher levels of VEGF-A, which not only stimulates tumor angiogenesis but also promotes macrophage survival [59]. TAMs, in turn, protect melanoma cells from apoptosis induced by the BRAF inhibitor vemurafenib [60]. Additionally, since MEK signaling in lymphocytes is crucial for T-cell activation and function, its inhibition has been shown to adversely affect early T-cell effector function [52]. Furthermore, immune alterations are commonly observed in MAPK inhibitor-resistant melanoma (Fig. 2). During the development of resistance to BRAF inhibitors, there is a marked reduction in highly activated inflammatory monocytes and dendritic cell subsets [61], specifically cDC1 (characterized by surface XCR1 staining) and cDC2 (characterized by surface CD11b staining). Additionally, a subpopulation expressing FcγRI/CD64 with elevated CD40 and CCR7 levels—indicative of their role in T cell-mediated tumor immunity—is diminished. This reduction, along with the accumulation of Treg cells, suggests a reversion of the tumor to an immunologically inert state [62]. Similarly, another study observed a TIME lacking functional CD103+ DCs in tumors when acquiring resistance to RAF inhibitors or RAF inhibitors in combination with MEK inhibitors, potentially leading to subsequent resistance to immunotherapy. Interestingly, this outcome is not only due to selective pressure from an immune response during the evolution of resistance but also arises from the tumor cell-intrinsic MAPK pathway itself. This pathway not only becomes reactivated but also demonstrates increased transcriptional output, driving immune evasion [63]. Another study revealed that tumor biopsies from patients with metastatic melanoma undergoing treatment with a BRAF inhibitor, who progressed on therapy, showed a significant decrease in melanoma antigen expression at the time of progression through reactivation of the MAPK pathway [51]. Consistently, loss of melanosomal antigens was observed in therapy-induced dedifferentiation, a reversible process characterized by the concomitant gain of neural crest markers and PD-L1 expression, reflecting a more invasive phenotype. This phenotypic plasticity has been observed with both BRAF inhibitors and adoptive T cell therapy resistance [64,65]. Intriguingly, this dedifferentiation signature may correlate with improved outcomes in anti-PD-1 therapy [[66], [67], [68]]. A high number of intratumoral CD163+ macrophages also correlate with BRAF inhibitor resistance. The transition from macrophages to CD163+ M2 macrophages was induced by exosome-derived growth factors and interleukins released by those resistant melanoma cells, Treg cells, and other macrophages [69]. Furthermore, a study tracking stage-specific changes in macrophages during BRAF/MEK inhibitor treatment in a melanoma model revealed that the emergence of a drug-tolerant persistent state coincided with a peak in the infiltration of Ccr2+ monocytes, known for their negative regulation of angiogenesis and T cell activity [[70], [71], [72]]. These Ccr2+ monocytes were also found to be key contributors in directing melanoma cell reprogramming towards specific therapeutic resistance pathways [70]. However, the precise mechanisms by which these drug-tolerant tumor cells recruit Ccr2+ monocytes remain to be elucidated.

In conclusion, immunomodulatory changes in the context of BRAF/MEK inhibitor resistance are characterized by decreased NK cell activation, M2 macrophage polarization driven by VEGF-A and tumor-derived factors, impaired T cell function, reduced dendritic cell infiltration (cDC1, cDC2, and CD40+CCR7+ subsets), Treg cell accumulation, and melanoma antigen loss due to MAPK reactivation and tumor dedifferentiation.

##### Cyclin-dependent kinase 4 and 6 inhibitor

The aberrant activation of the cyclin-dependent kinase 4 and 6 (CDK4/6) pathway, independent of mitogenic signaling, engenders uncontrolled breast cancer cell proliferation [73]. To date, three distinct CDK4/CDK6 inhibitors—palbociclib, ribociclib, and abemaciclib—have been approved for use in patients with hormone receptor-positive (HR+) breast cancer [[74], [75], [76]]. These inhibitors not only reduce the proliferation of cancer cells but also enhance the antitumor immune response. This is particularly noteworthy given that HR+ breast cancers are generally considered non-immunogenic compared to HER2-positive and triple-negative breast cancer (TNBC) tumors [61]. Palbociclib and abemaciclib mediate immunostimulatory effects by enhancing antigen presentation and promoting the secretion of pro-inflammatory cytokines. Abemaciclib increases MHC class I molecule exposure and induces antigen presentation signatures in breast cancer and colorectal cancer models [77,78]. Palbociclib enriches the MHC class I peptidome of melanoma cells with tumor-associated antigens and E2F target peptides [79]. CDK4/6 inhibitors also promote the secretion of pro-inflammatory cytokines, including interferons induced by tumor cell expression of endogenous retroviral elements, as well as CCL5 through an NF-κB-dependent mechanism [77,80]. Additionally, they directly interact with immune cells, leading to enhanced T cell activation partly through the de-repression of nuclear factor of activated T cells (NFAT) family proteins, and inhibition of Treg cells via the repression of DNA methyltransferase 1(DNMT1) [77,81]. Nevertheless, the immunostimulatory effects of CDK4/CDK6 inhibitors are partially counterbalanced by inducing speckle-type POZ (SPOP) protein deficiency. This deficiency disrupts the breakdown of interferon regulatory factor 1 (IRF1), leading to increased levels of PD-L1 [82].

Recently, deregulated immune pathways were found to be associated with CDK4/6 inhibitor resistance (Fig. 2). A recent study by Pandey et al. utilizing whole-exome sequencing and mRNA microarray analysis demonstrated that Palbociclib-resistant breast cancer cells exhibited upregulation of type I interferon (IFN) pathways and immune checkpoint inhibitory signals. Additionally, mutations in genes involved in immune regulation, such as NCOR1, MUC4, and MUC16, were identified in these resistant cells [83]. Similarly, another study demonstrated that aberrant IFN signaling pathway is associated with both intrinsic and acquired resistance to CDK4/6 inhibitors, coining the term “IFN-related Palbociclib-Resistance Signature” (IRPS) for the associated gene subset. They found that IRPS correlates with M1-polarized macrophages, Treg infiltration, and immune checkpoint expression. Although the M1 macrophage phenotype is classically associated with antitumor functions, the researchers speculated that chronic IFN signaling may alter the classical functions of M1 macrophages, potentially explaining worse long-term outcomes in patients with high IRPS [84]. Given that IFN can act as a double-edged sword, exerting both antitumor and protumor activities, and considering the high heterogeneity of ER+ breast cancer, which undergoes molecular and genetic alterations as the disease progresses [85], it is crucial to identify and characterize IFN signaling specific to each stage of the disease [86,87].

While deregulation of IFN signaling is a key feature of CDK4/6 inhibitor resistance, other immunomodulatory effects also exist, though they are less extensively studied. As previously mentioned, CDK4/6 inhibitors can elevate PD-L1 expression, thereby promoting immune evasion in breast cancer cell lines and mouse models. Additionally, certain senescence-associated secretory phenotypes (SASPs) induced by CDK4/6 inhibitors may contribute to local immunosuppression, including the recruitment of MDSCs [88]. In a separate study on HER2+ breast cancer, acquired resistance to CDK4/6 co-targeting therapy was associated with the presence of an immunosuppressive immature myeloid cell population resembling MDSCs. [89].

Overall, CDK4/6 inhibitor resistance is associated with aberrant IFN signaling (IRPS). Given the dual nature of IFN signaling, characterizing its specific role at each disease stage is crucial. Additional immunomodulatory effects, such as SASP-mediated MDSC recruitment, contribute to the development of an immunosuppressive microenvironment.

### Antibodies

#### EGFR antibody

EGFR antibodies, such as cetuximab, demonstrate superior clinical efficacy in colorectal cancer (CRC) compared to EGFR-TKIs, which are primarily used in non-small cell lung cancer (NSCLC) with activating EGFR kinase domain mutations. This disparity stems from their distinct mechanisms of action: EGFR antibodies effectively target EGFR overexpression regardless of mutational status, a characteristic feature of colorectal cancers [[90], [91], [92]]. Cetuximab is also frequently used in the treatment of HNSCC [93].

Unlike small molecule inhibitors that not directly linked to the activation of the immune response, monoclonal antibodies leverage distinct immune effector mechanisms, including antibody-dependent cellular cytotoxicity and phagocytosis (ADCC and ADCP) and complement-dependent cytotoxicity (CDC). ADCC and ADCP involve mAbs binding tumor antigens and engaging effector cells (NK cells, macrophages) via FcγRIIIa, triggering cytolysis or phagocytosis. CDC occurs when mAbs activate the complement cascade via C1q, forming the membrane-attack complex (MAC) and lysing the tumor cell. This complement activation also generates C3b, which opsonizes the tumor cell, further enhancing phagocytosis and cytolysis by macrophages and NK cells—a process known as complement-dependent cell-mediated cytotoxicity (CDCC) [94]. However, CDC in vivo is debated, as significantly higher mAb concentrations are required for CDC activation compared to ADCC, likely due to insufficient mAb surface density on target cells to activate the classical complement pathway [95].

**Fig. 3:**
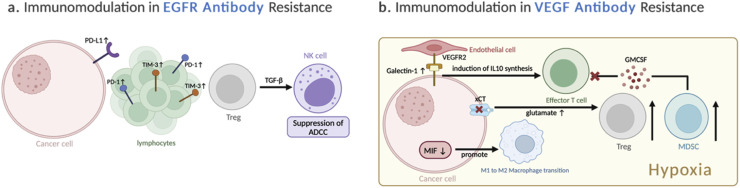
**Immunomodulation in Targeted Therapy Resistance – Antibodies** (a) Cetuximab resistance is linked to increased M2 macrophages, PD-L1 expression via epigenetic mechanisms, and upregulation of PD-1/TIM-3 on T cells. The observed increased Tregs suppress NK cell-mediated ADCC. (b) Adaptation to anti-VEGF therapy results in decreased MIF expression in tumor cells, ultimately driving macrophage polarization towards an M2 phenotype. Concurrently, persistent hypoxia fosters an immunosuppressive environment by increasing Treg suppression and MDSC recruitment, while also upregulating Galectin-1 to enhance angiogenesis and suppress T-cell responses. (ADCC: Antibody-Dependent Cell-Mediated Cytotoxicity; M2 Macrophages: Alternatively Activated Macrophages (M2 Phenotype); MIF: Macrophage Migration Inhibitory Factor; MDSC: Myeloid-Derived Suppressor Cells; NK cells: Natural Killer Cells; PD-1: Programmed cell death protein 1; PD-L1: Programmed Death-Ligand 1; TIM-3: T-cell immunoglobulin and mucin-domain containing-3; Tregs: Regulatory T cells; VEGF: Vascular Endothelial Growth Factor).

Notably, cetuximab has been shown to induce NK cell-mediated ADCC [88,89]. However, in cetuximab-opsonized CRC, NK cells are scarce while M2 macrophages are abundant, leading to the production of tumor-promoting factors like IL-10 and VEGF [96]. This may be related to a phenomenon observed after ADCP. Interestingly, cetuximab can also repolarize TAMs from M2-like to M1-like phenotypes by suppressing IL-6 expression through NF-κB and STAT3 pathways [97]. Furthermore, cetuximab can foster immunogenic cell death (ICD) in CRC cells, with an enhance in the endoplasmic reticulum stress response and an increase in phagocytosis by DC [98]. In cetuximab-resistant CRCs, an increase in M2 macrophages is observed, although the causality remains to be elucidated [99] (Fig. 3). Higher PD-L1 expression is evident in cetuximab-resistant HNSCC cell lines, despite no corresponding rise in *CD274* (which encodes PD-L1) mRNA levels, suggesting epigenetic modifications play a role in resistance [100]. Additionally, dynamic changes in PD-1 and TIM-3 expression on tumor-infiltrating lymphocytes during cetuximab treatment in HNSCC have been documented. [101]. Moreover, consistent cetuximab exposure increased Treg cells within the TIME, suppressing NK cell-mediated ADCC in a TGF-β1- dependent manner, leading to unfavorable outcomes [102].

In summary, while cetuximab can elicit ADCC and ICD, resistance development is associated with increased M2 macrophages, PD-L1 upregulation (likely epigenetic), elevated PD-1/TIM-3 expression on lymphocytes, and increased Treg infiltration, ultimately compromising its anti-tumor efficacy.

#### VEGF antibody

Targeting VEGF/VEGFR with specific antibodies has emerged as a promising strategy to both inhibit angiogenesis and enhance antitumor immunity. Nowadays, VEGF-A-targeting monoclonal antibody bevacizumab remains the most extensively characterized anti-angiogenetic treatment. Initially approved for treatment of metastatic CRC in combination with chemotherapy, its indications have expanded to include metastatic breast cancer, NSCLC, glioblastoma (GBM), RCC, hepatocellular carcinoma (HCC), ovarian cancer, and cervical cancer [103,104].

Unlike other cancer cell-targeted antibodies, anti-angiogenic antibodies are believed to primarily target the tumor's lifeline—its vascular system—focusing on endothelial cells. [105] This is supported by many studies that have failed to demonstrate significant direct cytotoxic effects of those antibodies [106,107]. Accordingly, these antibodies were found to mediate immunostimulatory effects independent of ADCC and ADCP [108]. Specifically, bevacizumab, alone or in combination with chemotherapy or radiation, has been associated with increased tumor infiltration by activated effective T cells coupled to Treg and TAM depletion [109], improved DC maturation [110], and downregulation of PD-L1 on malignant and myeloid cells in preclinical cancer models [111]. Similar findings have been documented in patients with NSCLC and ER-positive breast cancer treated with bevacizumab [112,113].

However, continuous exposure to anti-angiogenic agents can lead to tumor adaptation, involving a dynamic process that progresses from early-stage resistance primarily involving tumor cells, to later stages characterized by adaptation of the tumor microenvironment [46] (Fig. 3). Early when the treatment is still effective, bevacizumab reduces tumor cell MIF expression (MIF promotes M1 polarization, causing macrophages to be less phagocytic) that increased proliferation of TAMs at the tumor edge. Eventually, loss of MIF drives M1-to-M2 transition at the tumor edge and leads to disease progression [114]. Early response to anti-angiogenic therapy causes persistent intra-tumor hypoxia, leading to overexpression of galectin-1. This protein binds to VEGFR2 on endothelial cells, prolonging its surface presence and promoting angiogenesis independent of VEGF binding [115,116]. Galectin-1 has also been implicated in regulating immune responses by suppressing T cell proliferation and T cell-mediated inflammation through induction of IL-10 synthesis [117]. Similarly, hypoxia in GBM induced by anti-VEGF antibody exposure is shown to lead to dysfunctional glutamate/cystine antiporter (SLC7A11/xCT) activity of tumor cells. This in turn enhances Treg suppression via increased tumor-derived glutamate, fostering an immunosuppressive microenvironment [118]. Similar findings were observed in ovarian cancer, where bevacizumab-induced hypoxia upregulated GM-CSF levels in the tumor microenvironment. This promoted the migration and differentiation of MDSCs, leading to the inhibition of CD8+ lymphocyte proliferation [119].

To summarize, bevacizumab reduces MIF along with treatment adaption and thus promoting TAM proliferation and M2 polarization. Persistent hypoxia then upregulates galectin-1 (promoting angiogenesis and T cell suppression) and GM-CSF (driving MDSC accumulation and T cell inhibition), ultimately fostering an immunosuppressive microenvironment.

## Challenges in immunotherapy

### Efficacy of ICIs following targeted therapy: clinical evidence

#### EGFR-TKI

Several studies have demonstrated limited efficacy of ICIs in EGFR-mutant NSCLC after EGFR-TKI progression (Table 2). CheckMate 057 showed worse overall survival (OS) with nivolumab vs. docetaxel in EGFR-mutant patients (HR 1.18, 95% CI 0.69–2.00), unlike the OS benefit seen in wild-type EGFR patients (HR 0.66, 95% CI 0.51–0.86) [120]. Similarly, POPLAR found no OS benefit with atezolizumab vs. docetaxel in this subgroup (HR 0.99 vs. 0.70 in WT) [121,122]. KEYNOTE-010 also reported worse progression-free survival (PFS) with pembrolizumab vs. docetaxel in EGFR-mutant patients (HR 1.79, 95% CI 0.94–3.42), contrasting with improved PFS in wild-type EGFR patients (HR 0.83, 95% CI 0.71–0.98) [123]. More recently, CheckMate 722 showed no significant PFS improvement with nivolumab plus chemotherapy vs. chemotherapy alone in EGFR-mutant NSCLC after EGFR TKI progression (HR 0.75, 95% CI 0.56–1.00) [124]. Likewise, KEYNOTE-789 found that adding pembrolizumab to pemetrexed-platinum did not significantly improve PFS or OS vs. placebo in EGFR-mutant advanced NSCLC progressing on EGFR TKIs (PFS HR 0.80, 95% CI 0.65–0.97; OS HR 0.84, 95% CI, 0.69–1.02) [125]. Thus, both PD-L1 and PD-1 inhibition have failed to show benefit in EGFR-mutant patients in the second line setting. Efforts have been made to explore EGFR-TKI and ICI combinations. However, the phase III CAURAL trial (NCT02454933) evaluating osimertinib plus durvalumab vs. osimertinib monotherapy was prematurely terminated due to unacceptable interstitial lung disease in the combination arm, preventing definitive conclusions [126]. While the potential for synergy between EGFR-TKIs and ICIs warrants further investigation, robust clinical evidence remains limited.

**Table 2:** Summary of clinical studies demonstrating efficacy of ICIs following targeted therapy.

**Pathway**	**Study identifier**	**Cancer Type**	**Agents**	**Study design**	**Primary endpoint results**
**EGFR TKI**	**Check Mate 057** Phase III RCT	EGFRm advanced NSCLC failing EGFR TKI	Nivolumab Docetaxel	(A) Nivolumab (B) Docetaxel	OS HR 1.18 [95% CI 0.69–2.00]
	**Check Mate 722** Phase III trial	NSCLC failing 1st or 2nd line EGFR TKI	Nivolumab chemotherapy	(A) Nivolumab + chemotherapy (B) chemotherapy	PFS HR 0.75 [95% CI, 0.56–1.00]
	**KEYNOTE 010** Phase II/III RCT	EGFRm advanced NSCLC failing EGFR TKI	Pembrolizumab Docetaxel	(A) Pembrolizumab (B) Docetaxel	PFS HR 1.79 [95% CI 0.94–3.42]
	**POPLAR** Phase II RCT	EGFRm advanced NSCLC failing EGFR TKI	Atezolizumab Docetaxel	(A) Atezolizumab (B) Docetaxel	OS HR 0.99 [95% CI, 0.29–3.4]
	**KEYNOTE 789** Phase III RCT	EGFRm advanced NSCLC failing EGFR TKI	Pembrolizumab Chemotherapy	(A) Pembrolizumab + Chemotherapy (B) Placebo + Chemotherapy	PFS & OS HR 0.80 [95% CI, 0.65–0.97], *P=0.0122* HR 0.84 [95% CI, 0.69–1.02], *P = 0.0362* *(Efficacy boundary of PFS & OS: p= 0.0117 & 0.0118)*
**ALK-TKI**	**IMpower150** Phase III RCT	ALKr/t or EGFRm advanced NSCLC failing EGFR TKI	Atezolizumab Bevacizumab Carboplatin Paclitaxel	(A) Atezolizumab + Bevacizumab + Carboplatin + Paclitaxel (B) Bevacizumab + Carboplatin + Paclitaxel	PFS HR 0.59 [95% CI, 0.37- 0.94]
	**Jahanzeb *et.al***	ALKr/t advanced NSCLC	ALK TKIs ICIs	(A) ICI without prior ALK TKI (B) ICI with prior ALK TKI	PFS (A) 3.9 months [95% CI, 2.34, 8.59] (B) 1.5 months [95% CI, 1.18, 2.24]
**BRAF inhibitors**	**DREAMseq Trial** Phase III RCT	Metastatic BRAF^V600^ mutant melanoma	Ipilimumab Nivolumab dabrafenib trametinib	(A) Ipilimumab + Nivolumab induction, nivolumab maintenance; crossover to (C) upon PD (B) Dabrafenib + Trametinib; crossover to (D) upon PD (C) Dabrafenib + Trametinib (D) Ipilimumab + Nivolumab induction, nivolumab maintenance	2yr OS (A) 71.8% [95% CI, 62.5–79.1] (B) 51.5% [95% CI, 41.7–60.4] (log-rank p = 0.010)
	**SECOMBIT** Phase II trial	Metastatic BRAF^V600^ mutant melanoma	Ipilimumab Nivolumab Encorafenib Binimetinib	(A) Encorafenib + Binimetinib until PD, then Ipilimumab + Nivolumab (B) Ipilimumab + Nivolumab until PD, then Encorafenib + Binimetinib (C) Encorafenib + Binimetinib, then Ipilimumab + Nivolumab until PD, followed by Encorafenib + Binimetinib	2yr OS (A) 65% [95% CI, 54 to 76] (B) 73% [95% CI, 62 to 84] (C) 69% [95% CI, 59 to 80]
**Anti-angiogenic TKIs**	**Jing Li *et.al***	Advanced HCC	Sorafinib Lenvatinib Pembrolizumab Sintilimab	(A) Sorafinib or Lenvatinib + Pembrolizumab or Sintilimab (B) Sorafinib or Lenvatinib until PD, then added Pembrolizumab or Sintilimab	ORR (A) 25% (B) 4.3% *P=0.04*
**Anti-EGFR** **Antibodies**	**Checkmate 141** Phase III RCT	R/M HNSCC	Nivolumab Standard therapy	(A) Nivolumab versus ST with prior Cetuximab (B) Nivolumab versus ST without prior Cetuximab	OS (A) HR 0.84 [95% CI, 0.62–1.15] (A) HR 0.52 [ 95% CI, 0.35–0.77]
	**J.C.Park *et.al***	R/M HNSCC	Nivolumab	Nivolumab with prior Cetuximab Nivolumab without prior Cetuximab	OS HR 1.83 (*p= 0.031*)
**Anti-VEGF** **Antibodies**	**Tanimura *et.al***	Advanced NSCLC	Nivolumab Pembrolizumab	ICI with prior anti-VEGF antibodies ICI without prior anti-VEGF antibodies	PFS HR 1.83 [95% CI, 1.05–3.20]
	**Komiya *et.al***	Advanced NSCLC	Nivolumab	Nivolumab with prior anti-VEGF antibodies Nivolumab without prior anti-VEGF antibodies	PFS HR 1.44 [95% CI, 1.01–2.03]

(AEs: Adverse Events; ALK: Anaplastic Lymphoma Kinase; BRAF: B-Raf proto-oncogene, serine/threonine kinase; CI: Confidence Interval; DOR: Duration of Response; EGFRm: Epidermal Growth Factor Receptor Mutation; HCC: Hepatocellular Carcinoma; HNSCC: Head and Neck Squamous Cell Carcinoma; HR: Hazard Ratio; ICI: Immune Checkpoint Inhibitor; ITT: Intention-to-Treat; mo: Months; NSCLC: Non-Small Cell Lung Cancer; ORR: Objective Response Rate; OS: Overall Survival; PD: Progressive Disease; PFS: Progression-Free Survival; R/M: Recurrent/Metastatic; RCT: Randomized Controlled Trial; ST: Standard Therapy; TKI: Tyrosine Kinase Inhibitor; TPS: Tumor Proportion Score; VEGF: Vascular Endothelial Growth Factor)

#### ALK-TKI

Clinical trials investigating the benefits of ICIs after ALK-TKI treatment are much fewer than those following EGFR-TKI treatment, due to the lower prevalence of ALK translocations or rearrangements in NSCLC patients. IMpower150 is the phase III randomized controlled trial that includes a subgroup analysis of ALK-positive NSCLC patients [120]. This trial combined this small subgroup (representing only 3.3% of the total population) with patients harboring EGFR mutations. The result shows that adding atezolizumab to carboplatin, paclitaxel, and bevacizumab significantly improved PFS to 9.7 months compared to 6.1 months with chemotherapy plus bevacizumab alone. While OS data was not estimable, it also trended towards improvement in the atezolizumab group. However, the applicability of these findings specifically to ALK-positive NSCLC patients is limited due to the small sample size (n=13). On the other hand, a retrospective analysis have pointed to the relative lack of efficacy of ICI therapy in patients with ALK-positive NSCLC. In patients who received an ICI without prior ALK TKI, it was 3.9 months, and in patients who received ICI therapy after an ALK TKI, it was 1.5 months [121]. However, a concern with using ICI agents prior to ALK TKIs is the potential for increased toxicity. A real-world analysis revealed a significantly higher incidence of hepatotoxicity with sequential ICI treatment followed by crizotinib, compared to crizotinib alone, in ALK-positive NSCLC patients [122].

#### Anti-angiogenic MKI

A real-world practice in China is exploring the simultaneous and sequential use of anti-angiogenic MKIs plus ICIs in advanced HCC. Results indicate a higher objective response rate (ORR) in patients receiving simultaneous therapy compared to sequential therapy (25.0% vs. 4.3%, p=0.04). Although the simultaneous group showed trends toward improved survival outcomes, these differences did not reach statistical significance [123]. The ongoing phase 2 Seq-Cabo trial is investigating the sequential use of high-dose cabozantinib or cabozantinib plus nivolumab following progression on cabozantinib monotherapy in advanced RCC, which may offer further insights into this approach [124].

#### MAPK inhibitors

Both DREAMseq (phase III) and SECOMBIT (phase II) trials investigated treatment sequences in patients with untreated, BRAFV600-mutant metastatic melanoma. DREAMseq compared initial nivolumab/ipilimumab to dabrafenib/trametinib, with crossover upon progression, showing superior two-year OS for the initial nivolumab/ipilimumab group (71.8% vs 51.5%, log-rank p=0.010) [125]. SECOMBIT randomly assigned patients to three treatment arms: Arm A (encorafenib plus binimetinib until disease progression (PD) followed by ipilimumab plus nivolumab), Arm B (ipilimumab plus nivolumab until PD followed by encorafenib plus binimetinib), or Arm C (encorafenib plus binimetinib followed by ipilimumab plus nivolumab until PD, and then a return to encorafenib plus binimetinib). Two-year OS rates were also favorable across all arms, with Arm B showing the highest at 73% (95% CI, 62–84), followed by Arm C (69%; 95% CI, 59–80), and then Arm A (65%, 95% CI, 54–76) [126] These findings collectively suggest that initiating treatment with combination immunotherapy (nivolumab/ipilimumab) may be the preferred approach for patients with BRAFV600-mutant melanoma. Notably, the triple combination of atezolizumab (anti-PD-L1) with vemurafenib (BRAFi) and cobimetinib (MEKi) has received FDA approval for this population based on the phase III IMspire150 trial (NCT02908672), which demonstrated a significant improvement in PFS with the triplet therapy (15.1 months) compared to the doublet without ICI (10.6 months; HR, 0.78; 95% CI, 0.63–0.97) [127]. However, the phase III COMBI-i trial (NCT02967692) did not demonstrate a significant PFS benefit with the triple combination [128]. Given the conflicting PFS results from these phase III trials and the lack of direct comparisons between triplet therapy and upfront immunotherapy, further investigation is warranted to clarify the optimal treatment strategies.

#### CDK4/6 inhibitors

Preclinical and clinical trials investigating the combination of CDK4/6 inhibitors with ICIs for breast cancer suggest that combined use does not significantly enhance responses compared to CDK4/6 inhibitor monotherapy [129]. For example, the combination of abemaciclib with pembrolizumab in patients with HR+/HER2- metastatic breast cancer who had not previously received CDK4/6 inhibitors did not show a clear improvement compared to abemaciclib alone [130]. Similarly, combining palbociclib or ribociclib with a PD-1 inhibitor did not yield significant efficacy [131]. This lack of synergy, coupled with the high incidence of severe immune-related adverse events with combination therapy [132], has prompted interest in exploring sequential treatment strategies. While clinical data on ICI efficacy following CDK4/6 inhibitor exposure is currently limited, preclinical studies suggest that adaptation to CDK4/6 inhibitors may induce immunosuppressive effects, hindering immunotherapy responses. As previously mentioned, beyond the observed PD-L1 upregulation that is associated with intrinsic and acquired resistance to immunotherapy [133], sustained IFN signaling induced by CD4/6 blockade may also induce adaptive resistance through a PD-L1-independent multigenic program [84]. These findings highlight the intricate relationship between CDK4/6 inhibitors and the immune system, emphasizing the need for further research to optimize therapeutic sequencing strategies.

#### Anti-EGFR antibodies

The combination of ICIs with anti-EGFR antibodies has demonstrated the potential for synergistic anti-tumor activity [134]. However, the sequential administration of these therapeutic modalities may present challenges. In the phase III CheckMate 141 trial, nivolumab demonstrated improved overall OS compared to standard therapy in patients with recurrent/metastatic (R/M) HNSCC who progressed within 6 months of platinum-based chemotherapy. However, this benefit was more pronounced in patients without prior cetuximab exposure (HR 0.52; 95% CI, 0.35–0.77) compared to those with prior exposure (HR 0.84; 95% CI, 0.62–1.15) [135]. This trend was corroborated by a study by J.C. Park et al., also in R/M HNSCC, where prior cetuximab use was associated with worse OS (HR 1.83, *p*=0.031) in patients receiving subsequent ICI therapy [136].

#### Anti-VEGF antibodies

Tanimura et al. reported a significantly shorter PFS in patients with prior anti-VEGF exposure compared to those without (HR 1.83, 95% CI 1.05–3.20) in advanced NSCLC who received subsequent ICI therapy [137]. Similarly, Komiya et al. demonstrated a significant reduction in PFS with nivolumab in the prior anti-angiogenesis group (HR 1.44; 95% CI 1.01–2.03), with trends towards worse disease control and overall survival. These findings suggest a potential negative impact of prior anti-VEGF therapy on the efficacy of subsequent ICI treatment in this setting [138].

### Targeted therapy-induced TIME adaption and immunotherapy resistance

**Fig. 4:**
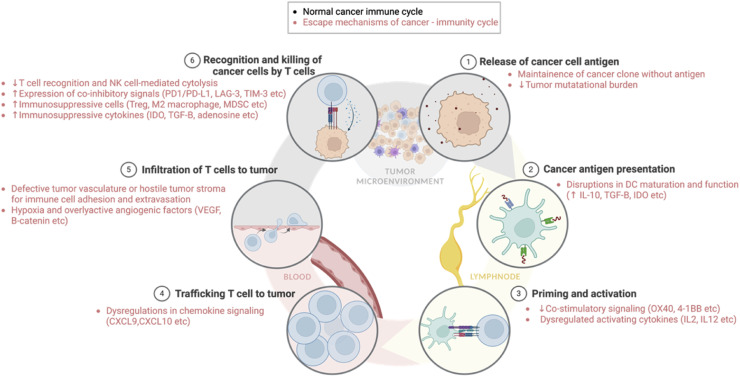
**Immune Escape Mechanisms in the Cancer-Immunity Cycle** (4–1BB: 4–1 Beta (also known as CD137); CXCL9: C-X-C Motif Chemokine Ligand 9; CXCL10: C-X-C Motif Chemokine Ligand 10; DC: Dendritic Cell; IDO: Indoleamine 2,3-Dioxygenase; IL-2: Interleukin-2; IL-10: Interleukin-10; IL-12: Interleukin-12; LAG-3: Lymphocyte-Activation Gene 3; MDSC: Myeloid-Derived Suppressor Cells; NK cell: Natural Killer cell; OX40: OX40 receptor (also known as CD134 or TNFRSF4); PD-1: Programmed cell death protein 1; PD-L1: Programmed death-ligand 1; TGF-β: Transforming Growth Factor-beta; TIM-3: T-cell immunoglobulin and mucin-domain containing-3; Treg: Regulatory T cell; VEGF: Vascular Endothelial Growth Factor).

The aforementioned immunomodulations resulting from target therapy adaptation are summarized in (Fig. 1, Fig. 2, Fig. 3). Notably, most of these adaptive microenvironments exhibit upregulation of PD-L1-staining cells. While in theory, subsequent anti-PD-L1/PD-1 blockade could successfully target these cells, it is generally accepted that the success of cancer immunotherapy hinges upon the effective orchestration of the cancer-immunity cycle (CI cycle), a series of interconnected steps crucial for generating anti-tumor immune responses. This cycle, operating within the framework of immunogenic cell death (ICD), necessitates the activation, mobilization, infiltration, viability, detection, and subsequent elimination of tumor cells by effector T cells [139] (Fig. 4). Escape at any stage of this cycle can limit anti-tumor immunity, thereby hindering tumor control [140]. Specifically, The failure of subsequent ICI therapy can be further contributed to two main categories of defects: (1) insufficient generation of anti-tumor T cells, and (2) inadequate function of tumor-specific T cells [141].

#### Insufficient generation of anti-tumor T cell

Successful ICI therapy relies on reactivating T cells that target tumor-specific mutant proteins [142]. The absence of appropriate neoantigens or alterations in antigen processing/presentation can hinder anti-tumor immune responses [143]. Adaptation to ALK-TKI downregulates antigen presentation genes (CALR, CANX, PDIA3) via unknown mechanisms, alongside reduced neoantigen load [51]. Similarly, BRAF inhibitor resistance decreases melanoma antigen expression owing to MAPK reactivation [33].

Adequate antigen-presenting cells are also required for generation of tumor-reactive CD8 T cells. Conventional dendritic cells (cDCs) play a major role, with cDC1s excelling at cross-presenting tumor antigens to CD8+ T cells [144,145]. While cDC2s primarily polarize CD4+ T helper cell responses, they also contribute to antitumor immunity and can present antigens to CD8+ T cells [[146], [147], [148]]. In a tumor regression model, CD11b+ cDC2 that displayed an IFN-stimulated gene expression (ISG+ DC) could present intact tumor-peptide-MHC-I complexes via cross-dressing [149]. During the development of resistance to BRAF inhibitors, a decrease in both cDC1 and cDC2 populations is observed, including a decline in highly activated, migratory cDC2s expressing FcγRI/CD64 in tumors and lymph nodes [62]. Further research substantiates that in MAPK inhibitor resistance, reactivated MAPK signaling in cancer cells drives immune evasion through enhanced transcriptional activity. This leads to low abundance and maturity of CD103+DCs, contributing to cross-resistance to immunotherapies [63]. Notably, this immune evasion is orchestrated by the cancer cells themselves and not a direct result of the targeted therapy affecting immune cells.

Impaired intratumoral T cell infiltration, along with inadequate APCs, also plays a significant role in ICI resistance. EGFR-TKI resistant cancer cells upregulate miR-21, leading to reduced pro-inflammatory cytokine release, hindering CD8+ T cell migration and impacting T cell differentiation [25]. Similarly, sunitinib resistance in RCC leads to elevated MDSCs through increased GM-CSF production, evading sunitinib-induced apoptosis [41]. These MDSCs generate peroxynitrite, which nitrates the T-cell receptors, reducing their responsiveness to antigen MHC complexes and T-cell specific chemokines, blocking T-cell migration [42].

#### Inadequate function of tumor-specific T cells

Following successful neoantigen presentation/cross-presentation and T-cell priming, the expanded pool of anti-tumor T cells encounters a hostile TIME that can impede their function, thus limiting the efficacy of ICI therapy [150,151]. These factors, both intrinsic and extrinsic to the tumor, encompass elevated PD-L1 expression on tumor and immune cells, high levels of alternate immune checkpoints or co-inhibitory receptors on T cells, and the presence of immunosuppressive cytokines or metabolites, along with the recruitment of immunosuppressive cells such as MDSCs and Treg [152].

Although PD-L1 upregulation is often associated with an improved response to ICIs, as it represents a compensatory mechanism following immune activation [153,154], PD-L1 can also be induced by cell-intrinsic signaling independent of the immune response to effector cells [155], further contributing the TIME to a “cold” state. This upward trend in PD-L1 expression is observed in multiple targeted-therapy resistant cancer cells. Notably, in EGFR-TKI resistant NSCLC, the MAPK, PI3K, and NF-κB pathways contribute to PD-L1 overexpression [26]. Similarly, PD-L1 expression is elevated in both the tumor and stromal compartments of ALK-TKI resistant tumors, although the exact mechanisms remain unclear [36]. In sunitinib-resistant RCC, upregulated RRM2 drives PD-L1 expression via the ANXA1/AKT signaling axis [48]. Additionally, CDK4/CDK6 inhibitors can induce a deficiency in SPOP protein, leading to the accumulation of the PD-L1 transcription factor IRF1, resulting in increased PD-L1 levels [82].

The abundance of Treg cells, Th2 cells, M2 TAMs, and MDSCs, which contribute to immunosuppression in the tumor microenvironment, poses a significant obstacle to immune therapies [[150], [151], [152]]. In EGFR-TKI resistant NSCLC, an increase in both IDO+ macrophages and Treg cells, capable of catabolizing tryptophan into immunosuppressive metabolites like kynurenine, is observed [24]. Notably, genes crucial for Treg function, such as SOCS1, are enriched in ceritinib-resistant tumors, aligning with the increased Treg presence [36]. Furthermore, adaptive hypoxia to anti-VEGF antibody leads to dysfunctional glutamate/cystine antiporter activity in tumor cells, elevating tumor-derived glutamate and subsequently enhancing Treg function [118]. Additionally, anti-VEGF antibody can upregulate both ANG2 and TIE2 expression, leading to increased infiltration of TEMs [44]. These TEMs suppress T cell proliferation, skew the CD4+/CD8+ T cell ratio, and promote the expansion of Tregs [45].

The generation of MDSCs is another hallmark of targeted therapy adaptation. Sorafenib-resistant pancreatic neuroendocrine tumors exhibit enrichment of PI3Kr CD11b+ immune cells (MDSCs), along with increased infiltration of pro-tumor Gr1+ monocytes and neutrophils [43]. Recruitment of MDSCs was seen in CDK4/6 inhibitors induced secretion of SASP by senescent cancer cells and fibroblast as well [43,88]. Macrophage repolarization towards a pro-tumor M2 phenotype is also observed. In BRAF inhibitor resistance, exosome-derived growth factors and interleukins released by resistant melanoma cells promote the transition of M2 macrophages [114]. Furthermore, constant exposure to bevacizumab reduce tumor cell MIF expression and inevitably drives M1-to-M2 transition [114].

## Strategies overcoming therapy-induced resistance to immunotherapy and future perspectives

**Fig. 5:**
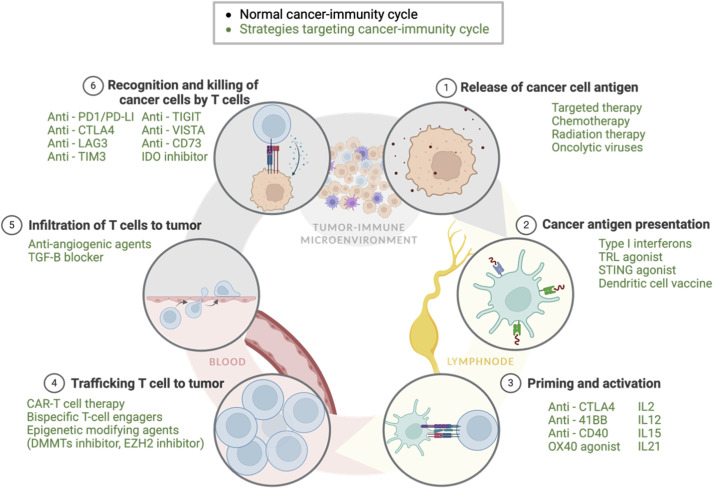
**Strategies Targeting the Cancer-Immunity Cycle** (ADCC: Antibody-Dependent Cell-Mediated Cytotoxicity; CAR-T cell: Chimeric Antigen Receptor T-cell; CTLA-4: Cytotoxic T-Lymphocyte-Associated protein 4; DC: Dendritic Cell; EZH2: Enhancer of zeste homolog 2; IDO: Indoleamine 2,3-Dioxygenase; IFN: Interferon; IL-2: Interleukin-2; IL-12: Interleukin-12; IL-15: Interleukin-15; LAG-3: Lymphocyte-Activation Gene 3; MHC: Major Histocompatibility Complex; NK cell: Natural Killer cell; OX40: OX40 receptor (also known as CD134 or TNFRSF4); PD-1: Programmed cell death protein 1; PD-L1: Programmed death-ligand 1; STING: Stimulator of Interferon Genes; TIGIT: T cell immunoreceptor with Ig and ITIM domains; TIM-3: T-cell immunoglobulin and mucin-domain containing-3; TGF-β: Transforming Growth Factor-beta; TRL: Toll-like Receptor; VISTA: V-domain Ig suppressor of T cell activation).

Addressing therapy-induced resistance to immunotherapy represents a significant challenge in contemporary oncology. In addition to administering immunotherapy prior to targeted therapy (with demonstrated clinical benefits shown in Table 2), other strategies aimed at mitigating immune escape mechanisms based on the CI cycle are briefly summarized below and in Fig. 5. Further details can be found in numerous excellent reviews [140,156,157] .

Enhancing tumor "immunogenicity"—the ability of a tumor to elicit an anti-tumor immune response—is often crucial for initiating anti-tumor immunity. Strategies that increase tumor antigen expression or stimulate the release of danger signals to promote immunogenic cell death (ICD), such as chemotherapy, targeted therapies, and radiotherapy, are fundamental to this goal and are core components of numerous FDA-approved combination regimens incorporating ICIs [156] . Subsequently, promoting neoantigen presentation is crucial. This can be achieved by stimulating innate immune responses and dendritic cell function through the utilization of type I interferons, TLR agonist, STING agonists and dendritic cell vaccine etc, as demonstrated by Pitt et al. and O'Donnell et al [150,152] . Furthermore, modulating inhibiting factors such as VEGF, IL-10, and TGF-β can significantly enhance dendritic cell migration, maturation, and function, thereby facilitating robust T-cell priming [150,152]. To further augment T-cell priming and activation, novel therapeutic approaches targeting T-cell co-stimulatory receptors, including OX40 (CD134) and 4–1BB (CD137), are being actively investigated, alongside the utilization of inflammatory cytokines (e.g., IL-2, IL-12, IL-15, IL-21) [158,159].

Successful T-cell trafficking and infiltration within the TIME necessitates addressing the complex immunosuppressive barriers. This involves the utilization of anti-angiogenic agents, and the implementation of adoptive cell therapies such as chimeric antigen receptor (CAR) T cells, and bispecific T-cell engagers (BiTEs) [157]. Moreover, epigenetic modifying agents (EMAs), including inhibitors of DNA methyltransferases (DNMTs) and histone methyltransferases (e.g., EZH2), have the potential to restore Th1 cytokine expression (e.g., CXCL9/10) and facilitate improved T-cell trafficking [160]

Given that ICIs primarily exert their therapeutic effects within the TIME, a significant focus of current research lies in reversing the escape mechanisms within this complex microenvironment. In addition to targeting CTLA-4 and PD-1 pathways, antibodies targeting other immune checkpoints (LAG-3, TIM-3, TIGIT, VISTA, etc.) are under active clinical and preclinical investigation [161] . Furthermore, therapeutic strategies aimed at depleting or inhibiting the activity of immunosuppressive cells, such as Tregs, TAMs, and MDSCs, as well as targeting immunosuppressive molecules like IDO inhibitors and anti-CD73 antibodies, are being actively explored [162] .

While many innovative therapies have emerged, anti-PD-1/PD-L1 therapy remains particularly prominent. Its success is likely attributable to the "Normalization Cancer Immunotherapy" concept, which focuses on restoring local immune homeostasis within the tumor-immune microenvironment. This approach contrasts with therapies like cytokine and anti-CTLA-4 therapies, which broadly enhance systemic immunity, increasing the risk of immune-related adverse events (irAEs) without necessarily addressing specific pre-existing immune deficiencies [163]. Those further highlight that the development of successful therapy relies on identifying specific defects in the antitumor immune response. These defects may exist intrinsic to the tumor, the TIME, or a reflection of patient genetics, microbiome, metabolism, or pharmacologic status but in each case must reflect the site of a rate limiting step in the CI cycle.

While classifying tumors by "immunotype" (e.g., immune-inflamed, -excluded, or -desert) may aid in selecting appropriate therapies to maximize anti-tumor responses (e.g., targeting TGF-β signaling to alter stromal architecture and permit T cell entry in preclinical models of immune-excluded tumors) [164], tumor immune escape mechanisms exhibit significant inter- and intra-tumor heterogeneity [165] . Encouragingly, next-generation histology has revolutionized our understanding of the TIME by enabling single-cell spatial analysis of both proteins and transcripts. Spatial proteomics, achieved through techniques like imaging mass cytometry [166], multiplexed ion beam imaging [167], and CODEX [168], offers unprecedented resolution of TIME heterogeneity. Similarly, histology-based transcriptomic platforms such as digital spatial profiling [169] and spatial transcriptomics [170] have illuminated new insights into the complex tumor-immune interface. By providing single-cell spatial resolution of the TIME, these techniques can reveal the heterogeneous landscape of resistance, uncovering multiple mechanisms that operate concurrently within the same tumor but are compartmentalized in distinct microregions and timeframes.

## Conclusion

In this article, we have explored the intricate immunomodulatory effects that arise with acquired resistance to targeted therapies, underscoring potential challenges for the efficacy of subsequent immunotherapy. However, the biology underlying immunomodulation by targeted anticancer agents remains in its early stages, with several key questions still unanswered. First, the historical development of targeted therapies has emphasized cell-autonomous mechanisms in immunodeficient preclinical models. Yet, these therapies exhibit robust immunomodulatory effects, raising questions about their target specificity and adding complexity to the immunomodulatory mechanisms in the context of targeted therapy resistance. Second, most targeted therapies display both immunostimulatory and immunoinhibitory effects, and the factors driving these divergent effects under specific conditions warrant further investigation. Third, although studies have reported alterations in the TIME upon resistance development, it remains unclear whether resistance precedes and drives these TIME changes, or if it is the stress induced by targeted therapy on the TIME that triggers resistance. These unresolved questions are crucial for understanding and addressing the reduced efficacy of subsequent immunotherapy, as current clinical research merely observes diminished effectiveness following targeted therapy without fully elucidating the underlying mechanisms. Further research is essential to optimize treatment combinations and sequencing strategies in this era of rapid advancements in both targeted therapies and immunotherapies.

## CRediT authorship contribution statement

**Ming-Yu Chou:** Writing – original draft, Visualization, Conceptualization. **Muh-Hwa Yang:** Writing – review & editing, Resources, Project administration, Funding acquisition, Conceptualization.

## Declaration of competing interest

The authors declare that they have no known competing financial interests or personal relationships that could have appeared to influence the work reported in this paper.
